# Resilient multi-agent RL: introducing DQ-RTS for distributed environments with data loss

**DOI:** 10.1038/s41598-023-48767-1

**Published:** 2024-01-23

**Authors:** Lorenzo Canese, Gian Carlo Cardarilli, Luca Di Nunzio, Rocco Fazzolari, Marco Re, Sergio Spanò

**Affiliations:** Department of Electronics, University of Rome Tor Vergata, 00133 Rome, Italy

**Keywords:** Electrical and electronic engineering, Information technology

## Abstract

This paper proposes DQ-RTS, a novel decentralized Multi-Agent Reinforcement Learning algorithm designed to address challenges posed by non-ideal communication and a varying number of agents in distributed environments. DQ-RTS incorporates an optimized communication protocol to mitigate data loss between agents. A comparative analysis between DQ-RTS and its decentralized counterpart Q-RTS, or Q-learning for Real-Time Swarms, demonstrates the superior convergence speed of DQ-RTS, achieving a remarkable speed-up factor ranging from 1.6 to 2.7 in scenarios with non-ideal communication. Moreover, DQ-RTS exhibits robustness by maintaining performance even when the agent population fluctuates, making it well-suited for applications requiring adaptable agent numbers over time. Additionally, extensive experiments conducted on various benchmark tasks validate the scalability and effectiveness of DQ-RTS, further establishing its potential as a practical solution for resilient Multi-Agent Reinforcement Learning in dynamic distributed environments.

## Introduction

Reinforcement Learning (RL) is a Machine Learning technique used to train an entity called “agent” to accomplish a particular task in a certain environment. The training of the agent is obtained through the maximization of a reward signal that represents a figure of merit depicting the effectiveness of the action taken by the agent. RL is an expanding sector that is found in a wide range of applications such as finance^1^, robotics^2–4^, natural language processing^5^, and communications^6^.

In recent years, a new sub-field of RL called Multi-Agent Reinforcement Learning (MARL) has found increasing interest in the literature^7,8^. In MARL, several agents interact with each other concurrently sharing the same environment. MARL generalizes and improves RL making it possible to accomplish more complex tasks in which several intelligent agents have to make decisions based on the action of the others. MARL has been proposed in several fields, for example, to model autonomous driving^9^, control fleets of drones^10^, telecommunications^11^, and energy sharing applications in smart grids^12^. The use of MARL is also desirable in IoT applications in which “IoT objects” have to operate in a distributed decentralized manner. In this context, MARL can be embedded directly into the items, thus forming an artificial swarm of agents.

In MARL, agents can interact with each other in 3 different settings: *cooperative, competitive, and mixed*. In a cooperative setting, all the agents receive a unique team reward based on their joint action. Agents are thus required to cooperate to solve the task, e.g. splitting their work into a series of more feasible sub-tasks. An example of this scenario is a fleet of drones equipped with a downwards-facing camera used to monitor and follow a moving target^10^. In competitive settings (also called zero-sum games), the sum of the rewards received by all agents is 0. An example of this scenario is the modeling of board games like chess or trading markets.

Mixed settings are a combination of the aforementioned in which agents exhibit some degree of cooperation and competition. An example of such a setting is the modeling of team games when the agent cooperates with their peers while competing against an adversary team. Lots of MARL algorithms capable of “super-human” performances in several scenarios have been presented^6,13–15^ in literature. Most of those algorithms have been proposed to operate on a traditional personal computer configuration (processor + GPU). The MARL algorithms presented in the literature mostly use independent agents that cannot communicate with each other^16^. In some cases, communication is possible through a central control center that does all the math for the agents^17^. In all these cases, there are significant limitations. Independent agents may fail to converge for cooperation tasks^18^; while a centralized coordinator implies a single point of failure if the central node is unavailable. To solve the aforementioned problem, we propose a novel MARL algorithm called Distributed Q-RTS (DQ-RTS) which is based on the multi-agent Q-RTS algorithm^19^. In DQ-RTS, agents exchange information between each others through a time varying communication network, this is similar to other works^20^ where message diffusion was used to train a fully decentralized multi-agent actor-critic. The difference is in the type of information exchanged observations or updated estimation as in our case and the structure of the algorithm executed by each agent. At the time of writing, this is the only MARL algorithm suitable for hardware-based implementations. The main innovation of the DQ-RTS algorithm is the capability for each agent to operate in a fully decentralized manner. This feature allows for the distribution of knowledge among the agents and the possibility to operate both in the case of failed data transmission and the variation of the number of agents.

## Background

Q-learning for Real-Time Swarm (Q-RTS)^19^ is a multi-agent generalization of Q-learning^21^ and it is meant for hardware-based implementations. It improves the convergence speed for real-time RL of intelligent swarms. Q-RTS allows for the sharing the swarm knowledge by using a *global swarm Q-matrix* $Q_{sw}$. The global matrix $Q_{sw}$ is computed by the central node that merges the N-agents *local Q-matrices* $Q_i$. The merging operation is carried out thanks to an aggregation function applied to the set $\Pi$ of all the local matrices (1).

1 $$\begin{aligned} Q_{sw}(s,a) = {\left\{ \begin{array}{ll} \underset{Q_i \in \Pi }{\max }\ Q_i(s,a), \quad \text{ if } \left|\underset{Q_i \in \Pi }{\max }\ Q_i(s,a) \right|> \left|\underset{Q_i \in \Pi }{\min }\ Q_i(s,a) \right|\\ \underset{Q_i \in \Pi }{\min }\ Q_i(s,a), \quad \text{ otherwise } \end{array}\right. } \end{aligned}$$

Each agent computes in parallel an *updated matrix* $Q'_i$ that is a linear combination of $Q_{sw}$ and $Q_i$. The agent evaluates its new local Q-matrix $Q_i$ by applying Q-learning update rule to $Q'_i$ (2). $\beta \in [0,1)$ is a parameter called *independence factor* which weights the local and global knowledge.

2 $$\begin{aligned} \left\{ \begin{aligned} Q_i(s_t,a_t)&\leftarrow (1-\alpha )Q'_i(s_t,a_t) + \alpha (r_i + \gamma \max _{\{ a \}} Q'_i(s_{t+1},a)) \\ Q'_i(s_t,a_t)&= \beta Q_i(s_t,a_t) + (1-\beta )Q_{sw}(s_t,a_t) \end{aligned}\right. \end{aligned}$$

## Distributed Q-RTS

We propose a novel fully decentralized MARL algorithm, inspired by Q-RTS. This method is optimized for swarm reinforcement learning applications, overcoming the above-discussed limitations due to communication issues with the main node.

For swarm applications, the use of a central node implies two main limitations:

- The design of the entities composing the swarm is heterogeneous as the central node is characterized by a different functionality with respect to the other agents.
- The central node represents a single point of failure: if this node fails, the correct behavior of the system is compromised.

The possibility of failed transmissions between agents and the central node is not considered in the literature. However, it is a very common event in some contexts, e.g. IoT wireless networks.

### Algorithm development

**Figure 1:**
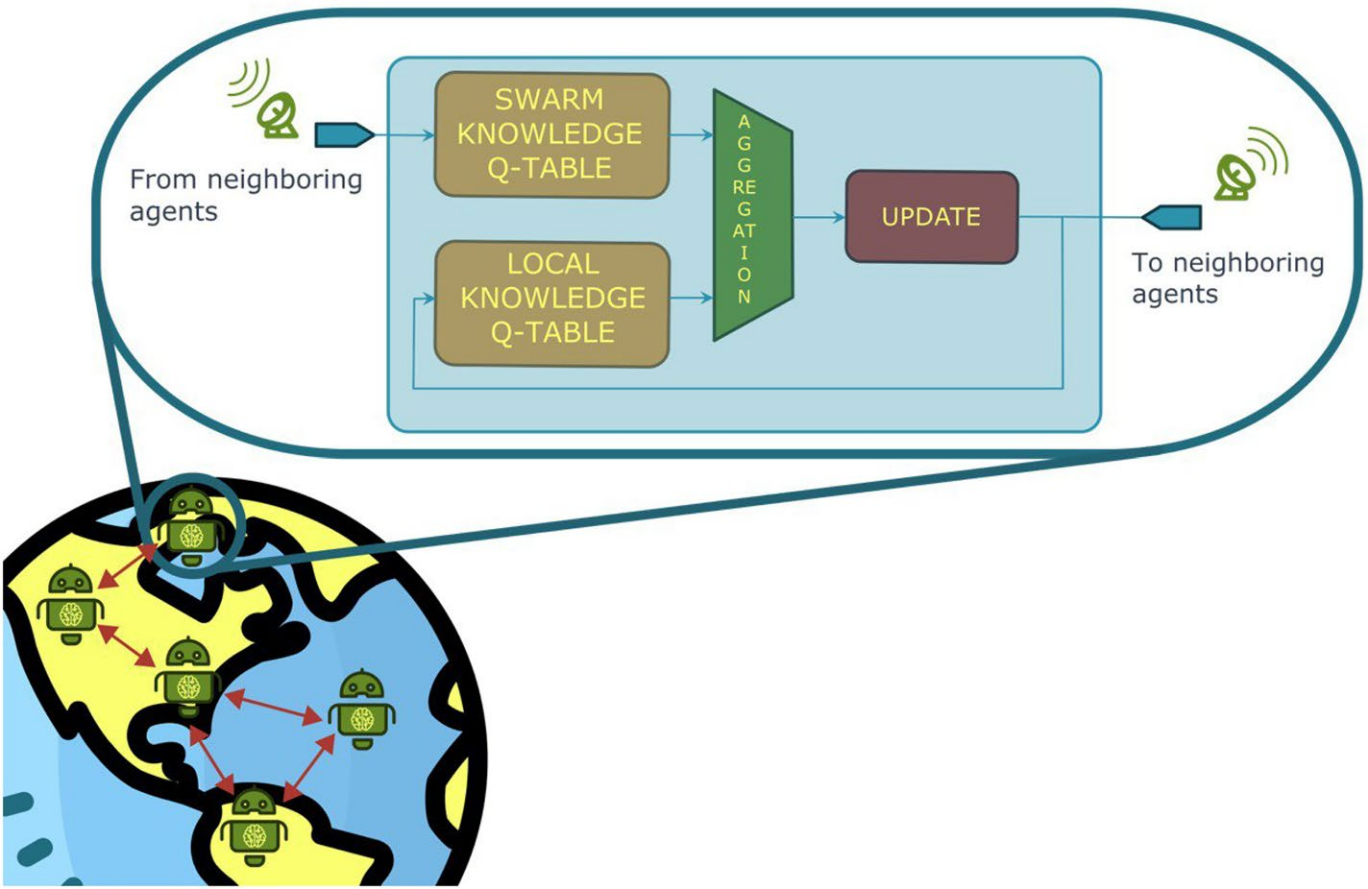
Structure of DQ-RTS algorithm. Each agent stores in its memory the two Q-Tables. The Swarm Q-table is updated using the information received from neighboring agents. The Local Q-table stores the matrix that the agent updates based on its experience. The two matrices are linearly combined and the result is used to perform the Q-learning update step.

To eliminate the need for a central node, DQ-RTS introduces a local swarm knowledge Q-matrix $Q^i_{sw}$ that is computed by each *i*-th agent. The algorithm operates in two phases: an *update phase* in which the agent interacts with the environment and updates its Q-table and a *communication phase* in which agents communicate with each other to share their knowledge. $Q^i_{sw}$ is computed in the latter phase. The algorithm estimates $Q_{sw}$ like Q-RTS but independently for each agent. A top-level overview of the algorithm can be found in fig 1. In the following, we analyze in detail the two algorithm phases.

#### Update phase

Before starting the learning process, each agent *i* initializes to zero its Q-table $Q^i$ and its swarm knowledge Q-table $Q^i_{sw}$ of size $|S |\times |A |$, where *S* is the number of states of the environment and *A* is the number of available actions. The training parameters are also initialized, the training rate $\alpha \in [0,1)$, the discount factor $\gamma \in [0,1)$, and the independence factor which is the weight used to combine the local and swarm Q matrix $\beta \in [0,1)$.

Each agent performs the update phase independently by following these steps:

1. Starting from the current state $s_t$, an action $a_t$ is selected in compliance with the chosen policy (for example an $\epsilon$-greedy policy).
2. The state is updated evolving from $s_t$ to $s_{t+1}$.
3. The agent receives a reward $r_{t}$.
4. The local Q-matrix $Q^i$ and the swarm knowledge Q-matrix $Q^i_{sw}$ of the agent *i* are locally combined, forming the update matrix $Q^i_{upd}$, according to the equation:
  3 $$\begin{aligned} Q^i_{upd} = \beta Q^i + (1 - \beta ) Q^i_{sw}. \end{aligned}$$
5. The Q-learning update rule is applied to $Q^i_{upd}$ to update the local Q-matrix.
  4 $$\begin{aligned} Q^i(s,a) = {\left\{ \begin{array}{ll} (1-\alpha ) Q^i_{upd}(s_t,a_t) + \alpha (r + \gamma \max _{a'} Q^i_{upd}(s_{t+1},a')) \; \text{ if } a = a_t\text { e }s = s_t\\ Q^i_{upd}(s,a) \quad \text{ otherwise } \end{array}\right. } \end{aligned}$$
6. $Q^i(s_t,a_t)$ is saved in the $Q^i_{sw}$ matrix if
  5 $$\begin{aligned} |Q^i(s_t,a_t) |\ge |Q^i_{sw}(s_t,a_t) |. \end{aligned}$$
7. The updated Q-value $Q^i(s_t,a_t)$ and its position index are transferred in a transmission buffer.
8. The communication phase starts.

#### Communication phase

In this phase, each agent sends and receives the Q-values to/from the other agents and then updates its swarm knowledge Q-Matrix $Q^i{sw}$. This is done by executing the following steps:

1. Each agent sends the messages saved in its transmission buffer to the other agents available.
2. The transmission buffer is cleaned.
3. Received messages are stored in a reception buffer. For each element in the buffer the following steps are executed:
  3.1. The Q-value $Q^j(s,a)$ received form the agent *j* is compared with the value in the local Q-matrix with the same index $Q^i(s,a)$.
  3.2. The swarm knowledge Q-matrix value $Q^i_{sw}(s,a)$ is updated by the following rule.
    6 $$\begin{aligned} Q^i_{sw}(s,a) = {\left\{ \begin{array}{ll} Q^i(s,a) \quad \text{ if } |Q^i(s,a) |> |Q^j(s,a) |\\ Q^j(s,a) \quad \text{ otherwise } \end{array}\right. } \end{aligned}$$
4. The reception buffer is cleaned.
5. The training time-step *t* is incremented and the agent moves to the next updating phase.

This procedure ensures that at the end of the communication phase each agent has stored in its swarm knowledge matrix $Q^i_{sw}$ the most important Q-values related to low and high reward signals. An overview of the above detailed phases is shown in Fig. 1.

### Agent communication

As discussed in the previous sections, in real-world applications the data transmission between the agents may fail for several reasons: connectivity problems, failure of one or more nodes, etc. In order to make the algorithm robust to these events, we developed a re-transmission protocol for the messages. It is supposed that the communication protocol is equipped with an acknowledgment mechanism and handshaking. In this way, every time an agent receives a message, it sends back an acknowledgment to confirm the correct receipt of the update.

The protocol’s operation mode is described in the following: for each agent, two vectors are defined. The first one, called *history vector* $\vec {H}$, contains all the state-action couples that the agent encountered during the learning. The second vector, called *Missed transmissions vector* $\vec {Mtx}$,contains the communication history. For example if we consider a case with 4 agents $\vec {Mtx}$ will be a vector with 4 elements. if the $\vec {Mtx}$ of the first agent 1 is [0, 12, 0, 5] it means that the agent has not communicated with agent 2 for 12 algorithm steps, 0 and 5 for respectively agents 3 and 4 (obviously the first element is always 0 since the agent do not communicate with himself). The state action couple is saved in $\vec {H}$ as a single integer representing the index of the Q-Matrix element related to the couple.

For each agent, the number of algorithm iterations passed since the last successful communication is stored. The elements of vector $\vec {H}$ are handled with First-In, First-Out (FIFO) methodology. So, during the update phase, the current state-action couple $(s_t,a_t)$ is stored in the FIFO and the first added element is deleted.

After the transmission there are two possibilities:

1. The agent does not receive an acknowledgment from agent *i* .
  1.1 The *ith* element of the Missed transmission vector is incremented.
    7 $$\begin{aligned} Mtx(i) = Mtx(i) + 1. \end{aligned}$$
2. The agent receives an acknowledgment from agent *j*
  2.1. The agent loads in its transmission buffer *Mtx*(*j*) the state-action couples (*s*, *a*) and the related Q-values from the local Q-matrix *Q*(*s*, *a*) from the most recent elements of the history vector $\vec {H}$.
  2.2. The update of the $Q_{sw}$ proceeds as described in the *Communication phase* section.
  2.3. *Mtx*(*j*) is set to zero since the agent does not have more missed messages to send to agent *j*.

**Figure 2:**
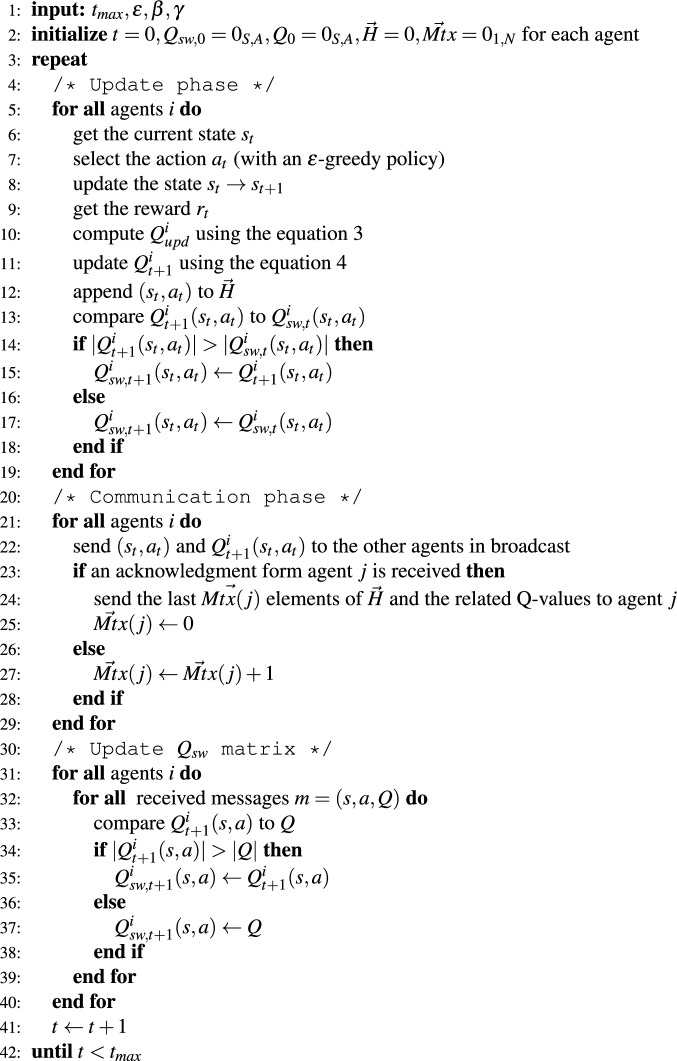
Pseudo-code for the DQ-RTS algorithm with limited range of communication.

The pseudo-code for the algorithm can be found in Fig. 2.

### Optimization of sent messages

We propose an optimization to limit the number of sent messages. If an agent interrupts the communication for a certain time, when the communication is reactivated it will have to send a high number of messages. This causes a communication overhead that could slow the training. During the communication failure, an agent can explore the same state-action couples more than once. However, for the training of the system, it is sufficient to know only if a state action couple has been explored and not how many times. For this reason, we adopted a data compression technique on the vector $\vec {H}$.

This compression technique works in this way:

- Each state-action couple is coded as an integer number and stored in a temporary vector called *temp*
- The elements of vector $\vec {temp}$ are sorted in ascending order
- The vector *temp2* is created by differentiating the values of temp as temp(i+1)-temp(i).
- We take the elements of *temp* that correspond to non-zero elements of *temp2*, that is we discard all zero-values of the differential.

**Figure 3:**
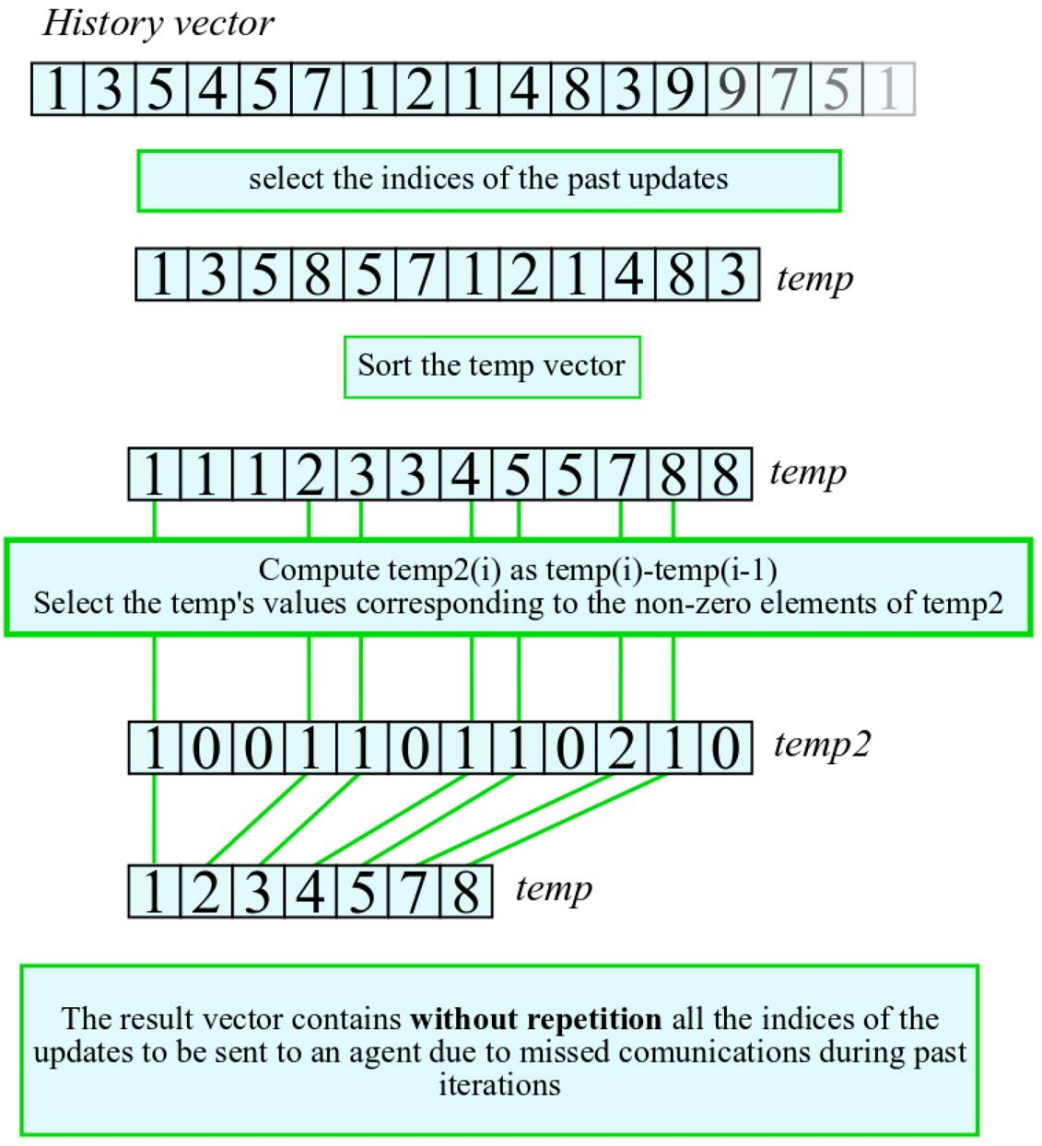
Method to reduce the number of messages to send.

This process is shown in Fig. 3.

### Robustness to change in the number of agents

As introduced before, DQ-RTS is also capable of operating in the case of a variation in the number of agents. If one or more agents are removed from the swarm (this is the case of damage or malfunction) there is no effect on the correct behavior of the algorithm. The only negative aspect is the slow-down of the convergence. Vice versa, if new agents are inserted into the swarm, these new agents receive the full information about the swarm matrix $Q_{sw}$ and they contribute to speed-up the convergence of the algorithm.

## Methods

To evaluate DQ-RTS and to estimate its performance, we performed the same test used in^19^ and compared the results. The evaluation environment was designed in MATLAB, it is a maze composed of cells, and each cell represents a state. So, the environment may be considered a grid. There are three cell types: free path, wall, and exit from the maze. Each agent can choose from among four actions, which are: move up, move down, move to the left and move to the right. The task of the agents is to reach the exit of the maze using the minimum number of steps. At each step of the algorithm, the agent receives a reward based on its choice. If the agent selects an action that will lead to a collision into a wall, it receives a large negative reward $r=-101$ and remains in its current state. However, if the agent moves to an accessible path (no collision with the wall) it receives a slight negative reward $r = -0.1$. Upon reaching the exit, the agent receives a positive reward $r = 100$, then it is moved to a random location and continues the training. This reward strategy was designed to motivate the agent to find the best path to exit the maze without collisions and in the least number of steps. To measure the performance we used two metrics. The first is the number of iterations required by the swarm to reach the optimal policy. Each maze has a single optimal solution that consists of the correct action to take for each state. The training is concluded when for each maze cell all the agents have learned the correct action to take. This metric indicates the time required by the swarm to converge (training time) to the optimal solution. The second metric is the one used in^19^. It is the average of the Q-values of the state before the maze’s exit over the simulation’s steps. If the value is small, the agents had collisions during the path while a high value indicates an absence of collisions. This metric shows how fast the agents can find the path to the exit. The training parameters were set as: $\alpha = 0.5$, $\beta = 0.1$, $\gamma = 0.9$ and both the swarm and the local Q-matrices were initialized to zero.

We performed two types of tests. In the first method, communication between the agents is ideal without any possibility of missed messages. In the second, we considered also communication problems by the agent, in particular, communication is possible for agents who are within a certain range from each other. This range is defined as the communication range and it is a simulation parameter. The test considering ideal communication was carried out for various maze sizes ($11 \times 11$, $15 \times 15$, $21 \times 21$, $31 \times 31$, $41 \times 41$) and the number of agents involved in the training (2, 4, 6, 8, 12, 16, 20, 24, 28). The goal is to demonstrate that DQ-RTS can achieve the same performance of Q-RTS when tested in the same scenario, with the benefits of a decentralized structure. The second test is oriented to estimate the performance of DQ-RTS, considering also the possibility of failed transmission. Experiments were carried out in the 31X31 maze with a different number of agents (2, 16, 24). We varied the communication span from a 15 cells radius (good communication range) to 2 (very poor communication range). Failed communication, in the Q-RTS algorithm, assumes a different meaning. It does not mean that two agents didn’t send their updates to each other, but that an agent failed to communicate with the central node. Thus, the agent does not receive the $Q_{sw}$ for that particular time step. In this case, the agent can not aggregate $Q_{sw}$ and $Q_{i}$. Thus, for the current time step, it will make an update using only the local Q matrix. This is equivalent to the traditional Q-learning, as it can be seen in Eq. (6) when $\beta = 1$. To determine the distance between the agents and the central node in the traditional Q-RTS counterpart, the latter was located in the center of the maze. In this way, the central node covers most of the area of the maze under its communication range. We show the simulations’ results in the following. We computed the mean and standard deviation of the chosen metrics over 50 simulations.

## Results and discussion

The results of the first experiment (ideal communication with unlimited range) are presented in Table 1. On the rows are shown the agents’ configuration, and in the columns, the mazes’ size. Results are expressed as the mean and standard deviation of algorithm convergence time. Considering several simulations.

Results confirm what is stated in the “Distributed Q-RTS” section. In the case of ideal communications, the performance of DQ-RTS and Q-RTS are equivalent, regardless of the size of the maze and the number of agents.

**Table 1:** DQ-RTS and Q-RTS convergence iterations comparison using different number of agents and maze size.

Maze size	$\pmb {11 \times 11}$	$\pmb {15 \times 15}$	$\pmb {21 \times 21}$	$\pmb {31 \times 31}$	$\pmb {41 \times 41}$
2 Agents DQ-RTS	$3195 \pm 371$	$16063 \pm 2150$	$60036 \pm 5799$	$29.975 \pm 3.139 \times 10^4$	$63.983 \pm 4.768 \times 10^4$
2 Agents Q-RTS	$3542 \pm 422$	$17703 \pm 2339$	$60616 \pm 6840$	$33.262 \pm 3.113 \times 10^4$	$71.067 \pm 4.768 \times 10^4$
4 Agents DQ-RTS	$1735 \pm 236$	$8640 \pm 1568$	$29081 \pm 2324$	$15.334 \pm 1.306 \times 10^4$	$33.504 \pm 2.615 \times 10^4$
4 Agents Q-RTS	$1812 \pm 252$	$8872 \pm 1110$	$30940 \pm 2983$	$16.728 \pm 1.521 \times 10^4$	$35.272 \pm 2.919 \times 10^4$
6 Agents DQ-RTS	$1217 \pm 166$	$5975 \pm 763$	$19890 \pm 1778$	$10.369 \pm 1.023 \times 10^4$	$22.372 \pm 2.187 \times 10^4$
6 Agents Q-RTS	$1239 \pm 158$	$5868 \pm 821$	$21096 \pm 2141$	$11.098 \pm 1.129 \times 10^4$	$24.523 \pm 2.414 \times 10^4$
8 Agents DQ-RTS	$971 \pm 134$	$4516 \pm 549$	$15210 \pm 1395$	$7.852 \pm 0.695 \times 10^4$	$16.670 \pm 1.353 \times 10^4$
8 Agents Q-RTS	$999 \pm 125$	$4572 \pm 638$	$15876 \pm 1551$	$8.320 \pm 0.564 \times 10^4$	$17.872 \pm 1.482 \times 10^4$
12 Agents DQ-RTS	$732 \pm 101$	$3151 \pm 555$	$10206 \pm 1118$	$5.160 \pm 0.458 \times 10^4$	$11.185 \pm 0.842 \times 10^4$
12 Agents Q-RTS	$667 \pm 83$	$3162 \pm 470$	$10779 \pm 1015$	$5.525 \pm 0.491 \times 10^4$	$12.073 \pm 1.130 \times 10^4$
16 Agents DQ-RTS	$574 \pm 74$	$2436 \pm 265$	$7820 \pm 771$	$3.928 \pm 0.318 \times 10^4$	$8.440 \pm 0.778 \times 10^4$
16 Agents Q-RTS	$555 \pm 57$	$2367 \pm 324$	$8228 \pm 756$	$4.212 \pm 0.325 \times 10^4$	$8.814 \pm 0.760 \times 10^4$
20 Agents DQ-RTS	$491 \pm 44$	$2091 \pm 186$	$6587 \pm 694$	$3.125 \pm 0.237 \times 10^4$	$6.682 \pm 0.587 \times 10^4$
20 Agents Q-RTS	$466 \pm 49$	$2040 \pm 210$	$6713 \pm 767$	$3.427 \pm 0.318 \times 10^4$	$7.257 \pm 0.622 \times 10^4$
24 Agents DQ-RTS	$439 \pm 46$	$1807 \pm 185$	$5547 \pm 434$	$2.624 \pm 0.204 \times 10^4$	$5.663 \pm 0.430 \times 10^4$
24 Agents Q-RTS	$388 \pm 40$	$1730 \pm 200$	$5678 \pm 405$	$2.892 \pm 0.297 \times 10^4$	$6.142 \pm 0.649 \times 10^4$
28 Agents DQ-RTS	$396 \pm 39$	$1807 \pm 167$	$4796 \pm 475$	$2.282 \pm 0.159 \times 10^4$	$4.789 \pm 0.396 \times 10^4$
28 Agents Q-RTS	$362 \pm 48$	$1500 \pm 166$	$4958 \pm 539$	$2.438 \pm 0.244 \times 10^4$	$5.206 \pm 0.436 \times 10^4$

Unlimited transmission radius range.

The results of the second experiment are shown in Table 2. As can be seen, for both algorithms, the convergence speed decreases as the communication range decreases.

**Table 2:** DQ-RTS and Q-RTS convergence iterations comparison using different number of agents and transmission radius range.

**Transmission radius**	**2 cells**	**4 cells**	**7 cells**	**10 cells**	**15 cells**
2 Agents Q-RTS	$410 \pm 16 \times 10^3$	$318 \pm 12 \times 10^3$	$215 \pm 16 \times 10^3$	$183 \pm 15 \times 10^3$	$166 \pm 17 \times 10^3$
2 Agents DQ-RTS	$253 \pm 12 \times 10^3$	$181 \pm 13 \times 10^3$	$165 \pm 13 \times 10^3$	$159 \pm 13 \times 10^3$	$153 \pm 11 \times 10^3$
16 Agents Q-RTS	$265 \pm 7 \times 10^3$	$164 \pm 5 \times 10^3$	$83 \pm 4 \times 10^3$	$61 \pm 4 \times 10^3$	$43 \pm 3 \times 10^3$
16 agents DQ-RTS	$111 \pm 3 \times 10^3$	$53 \pm 4 \times 10^3$	$44 \pm 3 \times 10^3$	$41 \pm 3 \times 10^3$	$39 \pm 3 \times 10^3$
28 Agents Q-RTS	$216 \pm 4 \times 10^3$	$130 \pm 3 \times 10^3$	$64 \pm 2 \times 10^3$	$44 \pm 2 \times 10^3$	$25 \pm 2 \times 10^3$
28 Agents DQ-RTS	$79 \pm 2 \times 10^3$	$32 \pm 1.2 \times 10^3$	$25 \pm 2 \times 10^3$	$25 \pm 2.3 \times 10^3$	$23 \pm 1.6 \times 10^3$

Maze size $$31 \times 31$$.

As the communication range decreases, DQ-RTS performs better than Q-RTS. This is because the presence of the hand-shake communication and the retransmission protocol makes it possible to retain the information related to states explored by the agent when it was isolated from the swarm.

The time required to reach the convergence is related to how fast an agent can communicate the information extracted during its exploration of the environment to every other agent. In Q-RTS, if the agent is too distant from the central node, the update is never recorded inside the swarm matrix. Thus, it will never be made available to other agents. Since in DQ-RTS each agent stores an estimation of $Q_{sw}$, it will share it with the rest of the swarm when it becomes available again. In other words, in a decentralized scheme, the distribution of knowledge among agents is more efficient.

In the DQ-RTS algorithm, the update of the swarm Q-matrix can be received either directly or indirectly. The first case is if two agents are inside the communication range and exchange Q-values. The second case exploits the distribution of the agents in the environment. Let us consider 3 agents A, B, and C with A into the communication range of B, and C into the communication range of B. A and C are not in their communication range. The Q-value sent from A to B in a given time step is used to update B’s Q-swam matrix. In the successive time steps, B sends to C Q-values dependent on B’s Q-swarm and local Q matrices. In such a way, the information obtained by A and sent to B is also received by C.

**Figure 4:**
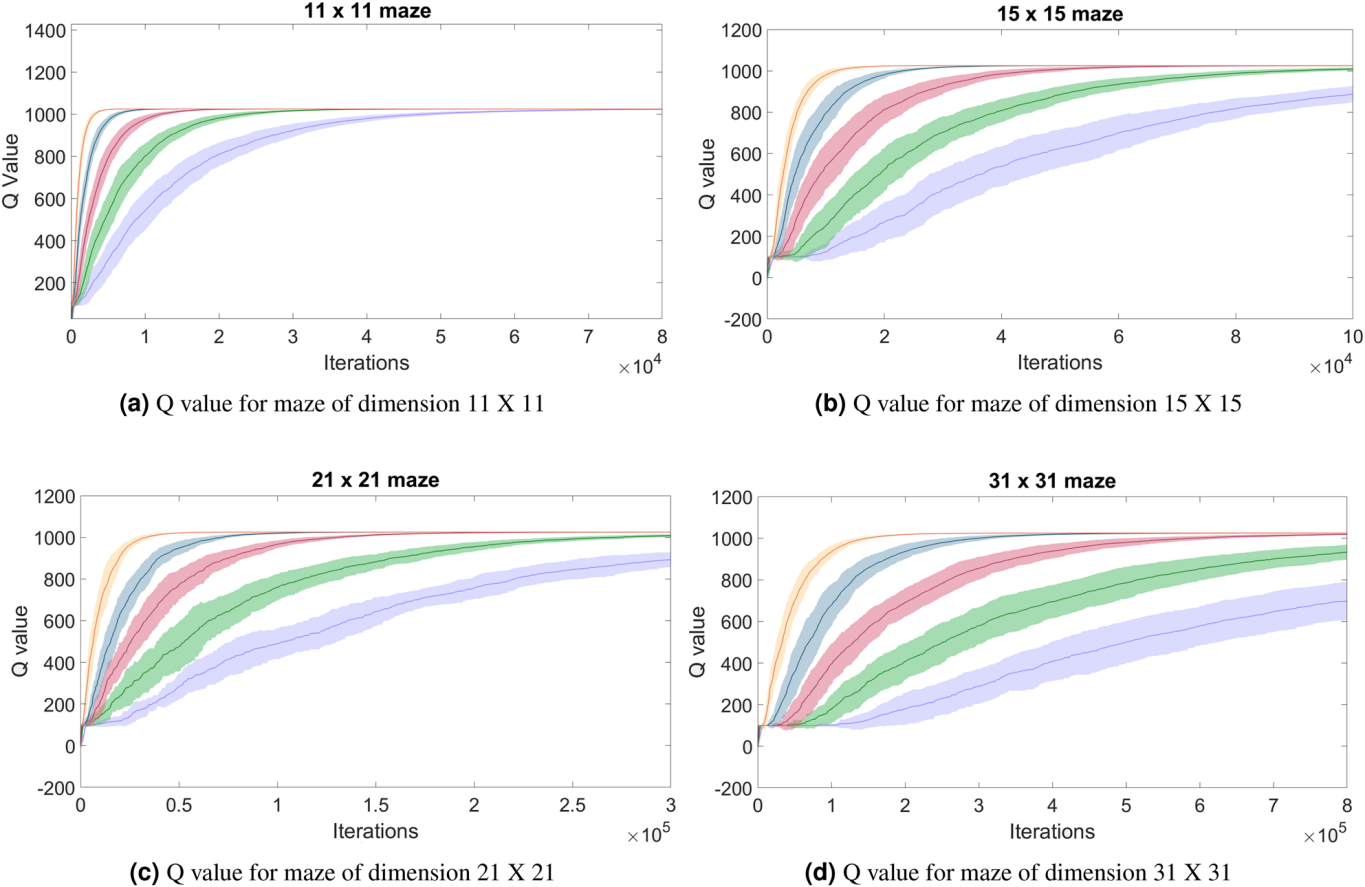
Each plot reports the Q-value computed by each agent when it exits the maze. 2 agents (purple), 4 agents (green), 8 agents (red), 16 agents (blue), 32 agents (yellow). The width represents the standard deviation, while the solid line the mean. Both quantities are computed over 50 simulations.

As shown in Table 2, DQ-RTS exhibits a lower performance reduction with the decrease in the communication range. When the range of communication covers the entire maze the performances are equivalent. For the minimum range of communication simulated (2 cells) DQ-RTS was 1.6 to 2.73 times faster in converging. The speed-up factor of 1.6 has been obtained considering 2 agents, while 2.7 has been obtained considering 28 agents. This is because if more agents share the same environment, they will communicate more often and distribute the information gained more efficiently. Fig. 4 shows that the capability of the agents to find the exit of the maze improves along with their number. Another interesting aspect is the decrease in the standard deviation with the increase in the number of agents.

From a performance point of view, DQ-RTS outperforms Q-RTS both in broadcast and local communication scenarios. However, it is important to note that the communication overhead of those algorithms is quite different. For each iteration, DQ-RTS sends a total of $N(N-1)$ updates packages, while Q-RTS just needs 2*N* updates message. The aforementioned retransmission protocol was used to reduce the negative effect of the added communication overhead. The effectiveness of the protocol is proportional to the sparsity of the communications between agents, and it manages to cut up to 60% of the communication overhead for the bigger environment with more distributed agents.

We presented the results in terms of the number of iterations needed to reach convergence. However, with a growing number of agents during each iteration, the number of computations that they have to perform increases too. Each update message received has to be compared to the local swarm table. The timing of the iteration depends mainly on the number of agents, as it increases with increasing agents. Iterations take less time if the communication network is more sparse, and that can lead to faster convergence times. Distributed computing for DQ-RTS, in comparison with the central paradigm of Q-RTS, requires less time to reach convergence with a typical speed-up factor in the range 4.5-5.5. Data and methods for the timing comparison can be found in the supplementary material (Supplementary Information).

### Discussion

DQ-RTS extends the implementability of Q-RTS to decentralized scenarios. This framework improves the robustness of the system by the elimination of the central aggregation node. Results show that in the presence of ideal communications, the performance of DQ-RTS and Q-RTS are equivalent (the swarm knowledge matrix is locally estimated by each agent). Vice-versa, in the case of real communication (with failures) DQ-RTS proved to be superior for every range of communication investigated. This is caused primarily by the use of the retransmission protocol, presented in the “Optimization of sent messages” section.

DQ-RTS outperforms Q-RTS since it is executed in parallel over a various number of agents. Q-RTS is executed on a single machine then the agent receives an update policy before taking the action. The time per iteration can be reduced by using a central node with more computational power, but that is not the case for edge computing. DQ-RTS is particularly fast in convergence time when the agent communicates sporadically; only relevant updates are sent when a communication appears, saving time. When the communication range decreases, the number of iterations to reach convergence increases. However, the time per iteration is reduced, resulting in overall faster convergence times. There is a trade-off between the sparsity of the network and the performance. When the network becomes too sparse, agents may fail to converge.

The messages between agents can cause a communication overhead. In this paper, solutions for this problem have not been investigated, but using a token system to transmit the update or to limit the number of agent-to-agent messages to a fixed number during the communication phase could be a solution.

**Figure 5:**
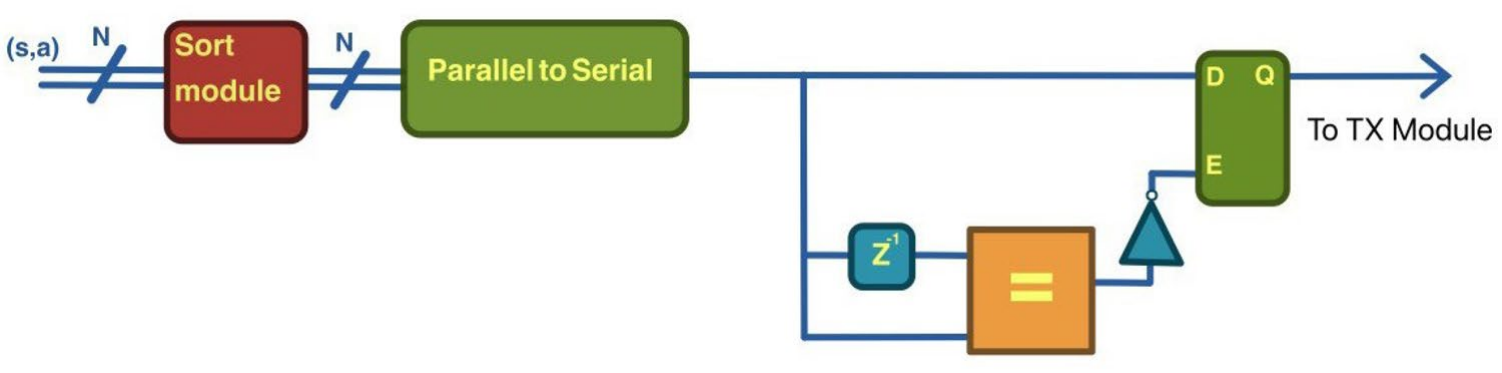
Possible hardware implementation for the selection of unique Q matrix indices inside of the history vector, the sorted values of the state-action couples in the transmission buffer are serialized, then using a delay block as a flip-flop we compare each value with the previous one and use the output of the comparison to select values to send to the trasmission module only when they differ, ensuring the unicity of the trasmitted update messages.

Another important aspect of the DQ-RTS is the possibility of being easily implemented in hardware digital circuits (such as Field Programmable Gate Array, FPGA) because it shares most of its structure with the Q-RTS that was fully implemented in FPGA^22^ and, at the moment of writing, it is the only FPGA-implementable MARL algorithm in the literature. In such a scenario, DQ-RTS could be implemented with minor modifications from Q-RTS. It is necessary to introduce two additional modules. A memory to be used for the storing of the past iterations and a circuit to select the values to be sent to each agent. A possible architecture is shown in Fig. 5.

### Supplementary Information

Supplementary Information.

## Data Availability

Reinforcement learning does not require training data, All the training was done in a simulator coded in MATLAB. For the code and the images of the mazes used in this paper contact author Canese Lorenzo.
