# Supplementary material for: Resilient multi-agent RL: introducing DQ-RTS for distributed environments with data loss
Source: Sci Rep. 2024 Jan 23;14:1994. doi: 10.1038/s41598-023-48767-1 (PMC10805896; doi:10.1038/s41598-023-48767-1)
Supplement: Supplementary file 1 — Supplementary Information. [file 41598_2023_48767_MOESM1_ESM.pdf]

Supplementary material

November 29, 2023

1 Time performance analysis

The method we used to estimate these times is the following: The algorithm is executed on a single machine simulating the network of agents. We measured the average time for iteration and divided it by the number of agents. This accounts for the computation time. To estimate the effect of the communication overhead, we counted the number of update messages sent by each agent and determined the time considering a package size of 92B and a realistic communication rate of 10 Mb/s In table 1, we present the average time per iteration at the variation of the number of agents and their range of communication. The time per iteration is highly dependent on both the number of agents and their range of communication. This is because when the number of agents rises, they will have to send more messages, and they need to process more update packets to estimate the swarm matrix. When agents communicate more sporadically due to reduced range, the time for each iteration becomes less.

We measured the actual time until convergence of DQ-RTS and Q-RTS with 28,16, and 2 agents in a 31×31 maze for various communication ranges. Results are presented on table 2. DQ-RTS clearly outperforms Q-RTS in every simulation, and the time to convergence decreases as the communication range is reduced. Q-RTS requires more time because the central node computes all the updates and sends them to the agents, while in DQ-RTS the computation load is divided in parallel among agents. Decreasing the communication range, the algorithm takes more steps to reach convergence. However, the time saved by not processing updates at each iteration made the convergence time lower. There is a limit for range reduction. Under a certain level, the agents are not able to exchange enough information to speed up the training process.

**Transmission radius**	**5 cells**	**7 cells**	**10 cells**	**no range**
**2 Agents**	17.2μs	18.1μs	18.5μs	23.7μs
**4 Agents**	19.2μs	19.6μs	21.7μs	34.9μs
**8 Agents**	21.8μs	24.1μs	32.2μs	66.4μs
**14 Agents**	27.2μs	29.9μs	39.3μs	92.1μs
**20 Agents**	29.4μs	37.5μs	49.9μs	125μs

Table 1: Time per iteration of DQ-RTS algorithm computed in a 31×31 mazes. It show the sum of computation time and communication time.

**Transmission radius**	**4 cells**	**7 cells**	**10 cells**	**no range**
**28 Agents Q-RTS**	4.00s	22.41s	19.34s	21.43s
**28 Agents DQ-RTS**	0.88s	1.036s	1.45s	4.05s
**16 Agents Q-RTS**	23.29s	20.03s	21.46s	36.07s
**16 Agents DQ-RTS**	4.33s	1.86s	1.51s	1.38s
**2 Agents Q-RTS**	20.42s	13.84s	11.90s	15.17s
**2 Agents DQ-RTS**	3.58s	3.08s	2.91s	3.37s

Table 2: Comparison of time to convergence of DQ-RTS and Q-RTS in the 31×31 maze for varying transmission ranges. Times decrease as the communication range decreases until a certain level; after that, times rise. This is the case of DQ-RTS for 16 agents when reducing the range from 4 to 2.
